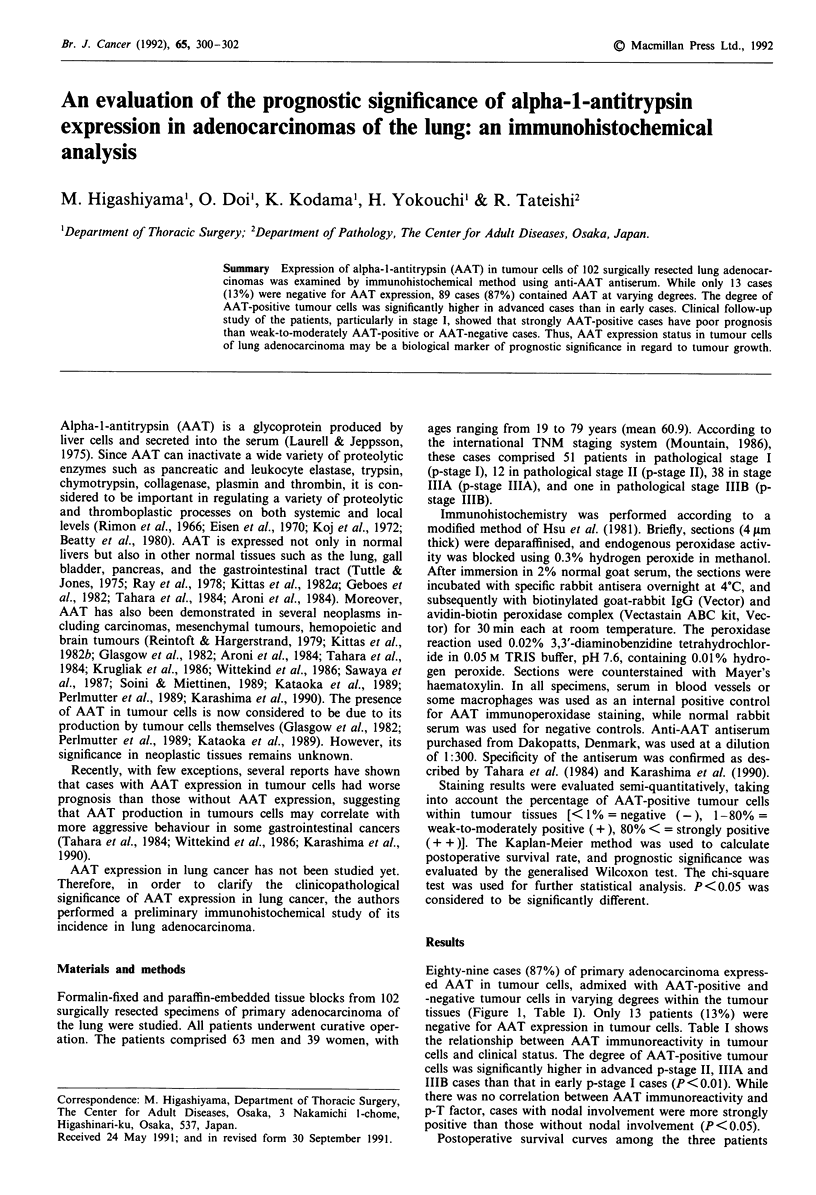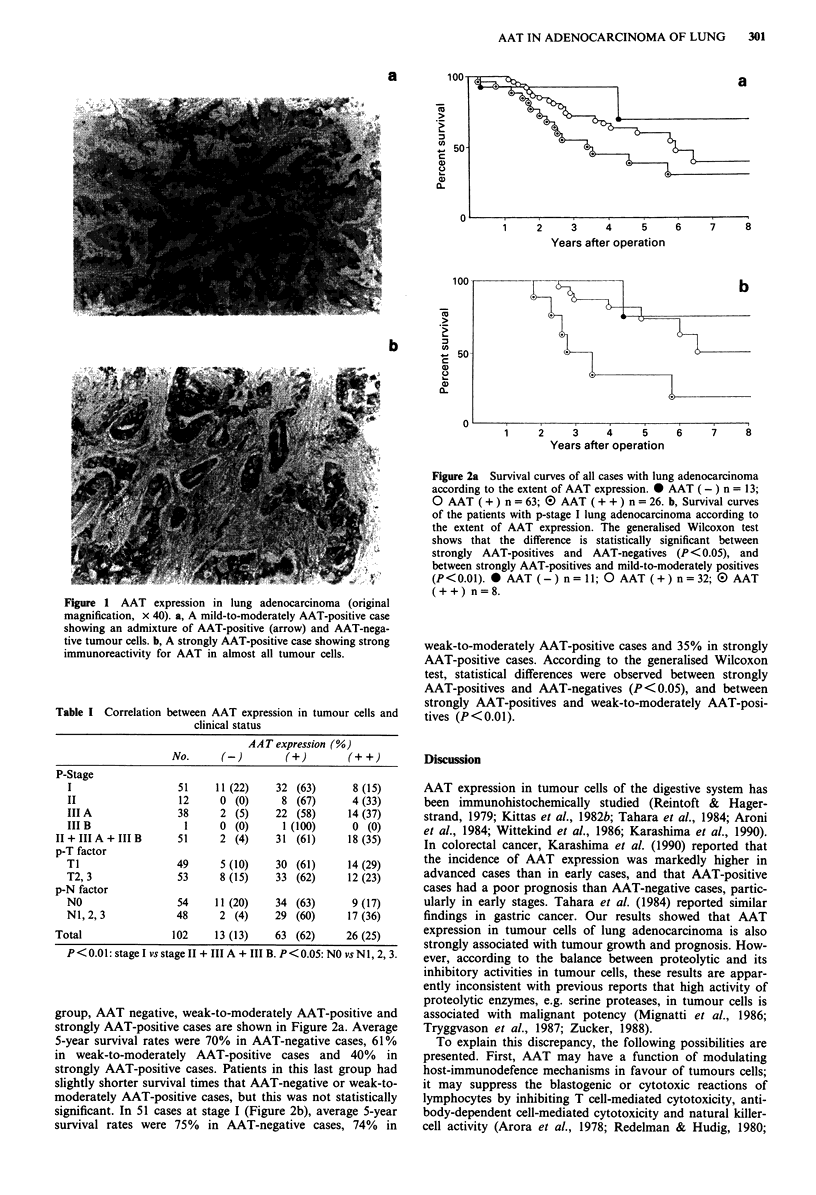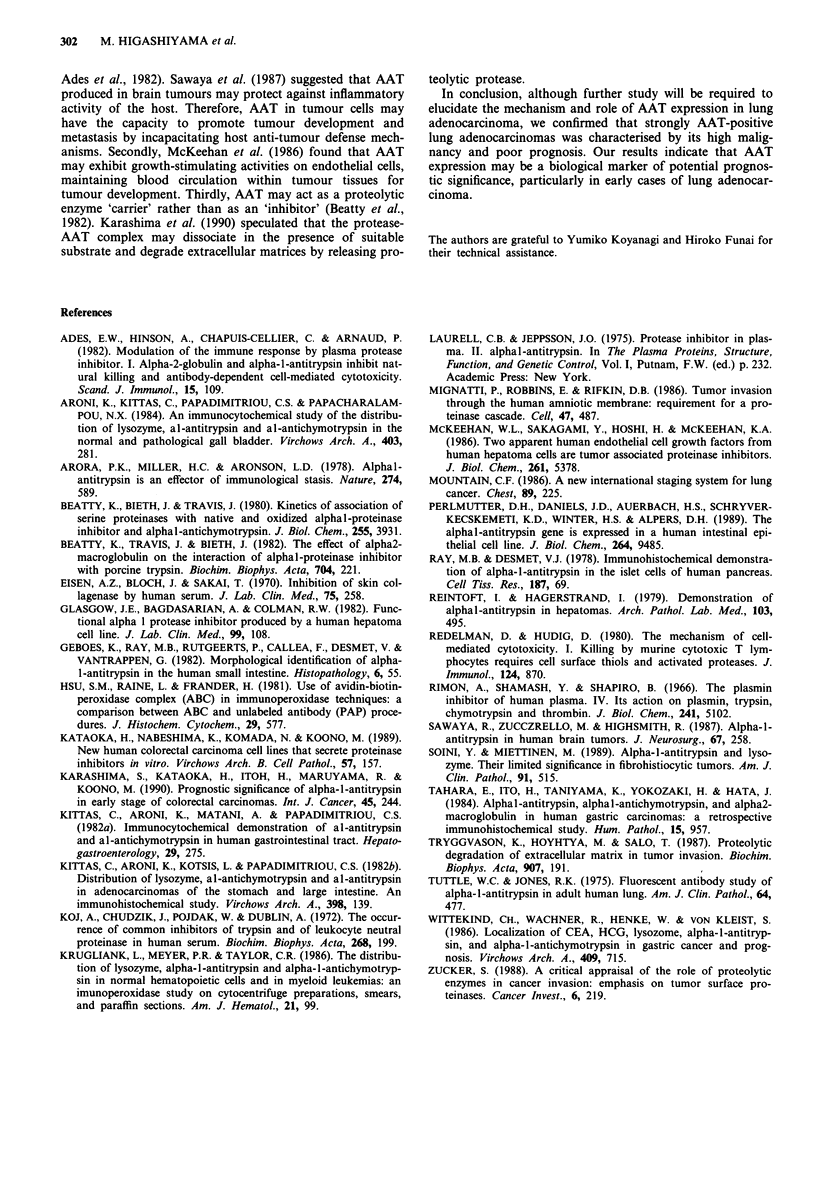# An evaluation of the prognostic significance of alpha-1-antitrypsin expression in adenocarcinomas of the lung: an immunohistochemical analysis.

**DOI:** 10.1038/bjc.1992.60

**Published:** 1992-02

**Authors:** M. Higashiyama, O. Doi, K. Kodama, H. Yokouchi, R. Tateishi

**Affiliations:** Department of Thoracic Surgery, Center for Adult Diseases, Osaka, Japan.

## Abstract

**Images:**


*Br. J. Cancer* (1992), **65**, 300–302 © Macmillan Press Ltd., 1992

### An evaluation of the prognostic significance of alpha-1-antitrypsin expression in adenocarcinomas of the lung: an immunohistochemical analysis

M. Higashiyama[1], O. Doi[1], K. Kodama[1], H. Yokouchi[1] & R. Tateishi[2]

[1]*Department of Thoracic Surgery;* [2]*Department of Pathology, The Center for Adult Diseases, Osaka, Japan.*

**Summary** Expression of alpha-1-antitrypsin (AAT) in tumour cells of 102 surgically resected lung adenocarcinomas was examined by immunohistochemical method using anti-AAT antiserum. While only 13 cases (13%) were negative for AAT expression, 89 cases (87%) contained AAT at varying degrees. The degree of AAT-positive tumour cells was significantly higher in advanced cases than in early cases. Clinical follow-up study of the patients, particularly in stage I, showed that strongly AAT-positive cases have poor prognosis than weak-to-moderately AAT-positive or AAT-negative cases. Thus, AAT expression status in tumour cells of lung adenocarcinoma may be a biological marker of prognostic significance in regard to tumour growth.

Alpha-1-antitrypsin (AAT) is a glycoprotein produced by liver cells and secreted into the serum (Laurell & Jeppsson, 1975). Since AAT can inactivate a wide variety of proteolytic enzymes such as pancreatic and leukocyte elastase, trypsin, chymotrypsin, collagenase, plasmin and thrombin, it is considered to be important in regulating a variety of proteolytic and thromboplastic processes on both systemic and local levels (Rimon *et al.*, 1966; Eisen *et al.*, 1970; Koj *et al.*, 1972; Beatty *et al.*, 1980). AAT is expressed not only in normal livers but also in other normal tissues such as the lung, gall bladder, pancreas, and the gastrointestinal tract (Tuttle & Jones, 1975; Ray *et al.*, 1978; Kittas *et al.*, 1982*a*; Geboes *et al.*, 1982; Tahara *et al.*, 1984; Aroni *et al.*, 1984). Moreover, AAT has also been demonstrated in several neoplasms including carcinomas, mesenchymal tumours, hemopoietic and brain tumours (Reintoft & Hargerstrand, 1979; Kittas *et al.*, 1982*b*; Glasgow *et al.*, 1982; Aroni *et al.*, 1984; Tahara *et al.*, 1984; Krugliak *et al.*, 1986; Wittekind *et al.*, 1986; Sawaya *et al.*, 1987; Soini & Miettinen, 1989; Kataoka *et al.*, 1989; Perlmutter *et al.*, 1989; Karashima *et al.*, 1990). The presence of AAT in tumour cells is now considered to be due to its production by tumour cells themselves (Glasgow *et al.*, 1982; Perlmutter *et al.*, 1989; Kataoka *et al.*, 1989). However, its significance in neoplastic tissues remains unknown.

Recently, with few exceptions, several reports have shown that cases with AAT expression in tumour cells had worse prognosis than those without AAT expression, suggesting that AAT production in tumours cells may correlate with more aggressive behaviour in some gastrointestinal cancers (Tahara *et al.*, 1984; Wittekind *et al.*, 1986; Karashima *et al.*, 1990).

AAT expression in lung cancer has not been studied yet. Therefore, in order to clarify the clinicopathological significance of AAT expression in lung cancer, the authors performed a preliminary immunohistochemical study of its incidence in lung adenocarcinoma.

### Materials and methods

Formalin-fixed and paraffin-embedded tissue blocks from 102 surgically resected specimens of primary adenocarcinoma of the lung were studied. All patients underwent curative operation. The patients comprised 63 men and 39 women, with ages ranging from 19 to 79 years (mean 60.9). According to the international TNM staging system (Mountain, 1986), these cases comprised 51 patients in pathological stage I (p-stage I), 12 in pathological stage II (p-stage II), 38 in stage IIIA (p-stage IIIA), and one in pathological stage IIIB (p-stage IIIB).

Immunohistochemistry was performed according to a modified method of Hsu *et al.* (1981). Briefly, sections (4 μm thick) were deparaffinised, and endogenous peroxidase activity was blocked using 0.3% hydrogen peroxide in methanol. After immersion in 2% normal goat serum, the sections were incubated with specific rabbit antisera overnight at 4°C, and subsequently with biotinylated goat-rabbit IgG (Vector) and avidin-biotin peroxidase complex (Vectastain ABC kit, Vector) for 30 min each at room temperature. The peroxidase reaction used 0.02% 3,3′-diaminobenzidine tetrahydrochloride in 0.05 M TRIS buffer, pH 7.6, containing 0.01% hydrogen peroxide. Sections were counterstained with Mayer's haematoxylin. In all specimens, serum in blood vessels or some macrophages was used as an internal positive control for AAT immunoperoxidase staining, while normal rabbit serum was used for negative controls. Anti-AAT antiserum purchased from Dakopatts, Denmark, was used at a dilution of 1:300. Specificity of the antiserum was confirmed as described by Tahara *et al.* (1984) and Karashima *et al.* (1990).

Staining results were evaluated semi-quantitatively, taking into account the percentage of AAT-positive tumour cells within tumour tissues [$<1\%$ = negative (−), 1–80% = weak-to-moderately positive (+), $80\% <=$ strongly positive (++)]. The Kaplan-Meier method was used to calculate postoperative survival rate, and prognostic significance was evaluated by the generalised Wilcoxon test. The chi-square test was used for further statistical analysis. $P<0.05$ was considered to be significantly different.

### Results

Eighty-nine cases (87%) of primary adenocarcinoma expressed AAT in tumour cells, admixed with AAT-positive and -negative tumour cells in varying degrees within the tumour tissues (Figure 1, Table I). Only 13 patients (13%) were negative for AAT expression in tumour cells. Table I shows the relationship between AAT immunoreactivity in tumour cells and clinical status. The degree of AAT-positive tumour cells was significantly higher in advanced p-stage II, IIIA and IIIB cases than that in early p-stage I cases ($P<0.01$). While there was no correlation between AAT immunoreactivity and p-T factor, cases with nodal involvement were more strongly positive than those without nodal involvement ($P<0.05$).

Postoperative survival curves among the three patients

Correspondence: M. Higashiyama, Department of Thoracic Surgery, The Center for Adult Diseases, Osaka, 3 Nakamichi 1-chome, Higashinari-ku, Osaka, 537, Japan.
Received 24 May 1991; and in revised form 30 September 1991.

**Figure 1** AAT expression in lung adenocarcinoma (original magnification, × 40). **a**, A mild-to-moderately AAT-positive case showing an admixture of AAT-positive (arrow) and AAT-negative tumour cells. **b**, A strongly AAT-positive case showing strong immunoreactivity for AAT in almost all tumour cells.

**Figure 2a** Survival curves of all cases with lung adenocarcinoma according to the extent of AAT expression. ● AAT ( – ) n = 13; O AAT ( + ) n = 63; ⊙ AAT ( + + ) n = 26. **b**, Survival curves of the patients with p-stage I lung adenocarcinoma according to the extent of AAT expression. The generalised Wilcoxon test shows that the difference is statistically significant between strongly AAT-positives and AAT-negatives ($P<0.05$), and between strongly AAT-positives and mild-to-moderately positives ($P<0.01$). ● AAT ( – ) n = 11; O AAT ( + ) n = 32; ⊙ AAT ( + + ) n = 8.

**Table I** Correlation between AAT expression in tumour cells and clinical status

| | | *AAT expression (%)* | | |
|---|---|---|---|---|
| | *No.* | *( – )* | *( + )* | *( + + )* |
| P-Stage | | | | |
| I | 51 | 11 (22) | 32 (63) | 8 (15) |
| II | 12 | 0 (0) | 8 (67) | 4 (33) |
| III A | 38 | 2 (5) | 22 (58) | 14 (37) |
| III B | 1 | 0 (0) | 1 (100) | 0 (0) |
| II + III A + III B | 51 | 2 (4) | 31 (61) | 18 (35) |
| p-T factor | | | | |
| T1 | 49 | 5 (10) | 30 (61) | 14 (29) |
| T2, 3 | 53 | 8 (15) | 33 (62) | 12 (23) |
| p-N factor | | | | |
| N0 | 54 | 11 (20) | 34 (63) | 9 (17) |
| N1, 2, 3 | 48 | 2 (4) | 29 (60) | 17 (36) |
| Total | 102 | 13 (13) | 63 (62) | 26 (25) |

$P<0.01$: stage I *vs* stage II + III A + III B. $P<0.05$: N0 *vs* N1, 2, 3.

group, AAT negative, weak-to-moderately AAT-positive and strongly AAT-positive cases are shown in Figure 2a. Average 5-year survival rates were 70% in AAT-negative cases, 61% in weak-to-moderately AAT-positive cases and 40% in strongly AAT-positive cases. Patients in this last group had slightly shorter survival times that AAT-negative or weak-to-moderately AAT-positive cases, but this was not statistically significant. In 51 cases at stage I (Figure 2b), average 5-year survival rates were 75% in AAT-negative cases, 74% in weak-to-moderately AAT-positive cases and 35% in strongly AAT-positive cases. According to the generalised Wilcoxon test, statistical differences were observed between strongly AAT-positives and AAT-negatives ($P<0.05$), and between strongly AAT-positives and weak-to-moderately AAT-positives ($P<0.01$).

### Discussion

AAT expression in tumour cells of the digestive system has been immunohistochemically studied (Reintoft & Hagerstrand, 1979; Kittas *et al.*, 1982*b*; Tahara *et al.*, 1984; Aroni *et al.*, 1984; Wittekind *et al.*, 1986; Karashima *et al.*, 1990). In colorectal cancer, Karashima *et al.* (1990) reported that the incidence of AAT expression was markedly higher in advanced cases than in early cases, and that AAT-positive cases had a poor prognosis than AAT-negative cases, particularly in early stages. Tahara *et al.* (1984) reported similar findings in gastric cancer. Our results showed that AAT expression in tumour cells of lung adenocarcinoma is also strongly associated with tumour growth and prognosis. However, according to the balance between proteolytic and its inhibitory activities in tumour cells, these results are apparently inconsistent with previous reports that high activity of proteolytic enzymes, e.g. serine proteases, in tumour cells is associated with malignant potency (Mignatti *et al.*, 1986; Tryggvason *et al.*, 1987; Zucker, 1988).

To explain this discrepancy, the following possibilities are presented. First, AAT may have a function of modulating host-immunodefence mechanisms in favour of tumours cells; it may suppress the blastogenic or cytotoxic reactions of lymphocytes by inhibiting T cell-mediated cytotoxicity, antibody-dependent cell-mediated cytotoxicity and natural killer-cell activity (Arora *et al.*, 1978; Redelman & Hudig, 1980;

Ades *et al*., 1982). Sawaya *et al*. (1987) suggested that AAT produced in brain tumours may protect against inflammatory activity of the host. Therefore, AAT in tumour cells may have the capacity to promote tumour development and metastasis by incapacitating host anti-tumour defense mechanisms. Secondly, McKeehan *et al*. (1986) found that AAT may exhibit growth-stimulating activities on endothelial cells, maintaining blood circulation within tumour tissues for tumour development. Thirdly, AAT may act as a proteolytic enzyme 'carrier' rather than as an 'inhibitor' (Beatty *et al*., 1982). Karashima *et al*. (1990) speculated that the protease-AAT complex may dissociate in the presence of suitable substrate and degrade extracellular matrices by releasing proteolytic protease.

In conclusion, although further study will be required to elucidate the mechanism and role of AAT expression in lung adenocarcinoma, we confirmed that strongly AAT-positive lung adenocarcinomas was characterised by its high malignancy and poor prognosis. Our results indicate that AAT expression may be a biological marker of potential prognostic significance, particularly in early cases of lung adenocarcinoma.

The authors are grateful to Yumiko Koyanagi and Hiroko Funai for their technical assistance.

### References

ADES, E.W., HINSON, A., CHAPUIS-CELLIER, C. & ARNAUD, P. (1982). Modulation of the immune response by plasma protease inhibitor. I. Alpha-2-globulin and alpha-1-antitrypsin inhibit natural killing and antibody-dependent cell-mediated cytotoxicity. *Scand. J. Immunol*., **15**, 109.

ARONI, K., KITTAS, C., PAPADIMITRIOU, C.S. & PAPACHARALAMPOU, N.X. (1984). An immunocytochemical study of the distribution of lysozyme, a1-antitrypsin and a1-antichymotrypsin in the normal and pathological gall bladder. *Virchows Arch. A*., **403**, 281.

ARORA, P.K., MILLER, H.C. & ARONSON, L.D. (1978). Alpha1-antitrypsin is an effector of immunological stasis. *Nature*, **274**, 589.

BEATTY, K., BIETH, J. & TRAVIS, J. (1980). Kinetics of association of serine proteinases with native and oxidized alpha1-proteinase inhibitor and alpha1-antichymotrypsin. *J. Biol. Chem*., **255**, 3931.

BEATTY, K., TRAVIS, J. & BIETH, J. (1982). The effect of alpha2-macroglobulin on the interaction of alpha1-proteinase inhibitor with porcine trypsin. *Biochim. Biophys. Acta*, **704**, 221.

EISEN, A.Z., BLOCH, J. & SAKAI, T. (1970). Inhibition of skin collagenase by human serum. *J. Lab. Clin. Med*., **75**, 258.

GLASGOW, J.E., BAGDASARIAN, A. & COLMAN, R.W. (1982). Functional alpha 1 protease inhibitor produced by a human hepatoma cell line. *J. Lab. Clin. Med*., **99**, 108.

GEBOES, K., RAY, M.B., RUTGEERTS, P., CALLEA, F., DESMET, V. & VANTRAPPEN, G. (1982). Morphological identification of alpha-1-antitrypsin in the human small intestine. *Histopathology*, **6**, 55.

HSU, S.M., RAINE, L. & FRANDER, H. (1981). Use of avidin-biotin-peroxidase complex (ABC) in immunoperoxidase techniques: a comparison between ABC and unlabeled antibody (PAP) procedures. *J. Histochem. Cytochem*., **29**, 577.

KATAOKA, H., NABESHIMA, K., KOMADA, N. & KOONO, M. (1989). New human colorectal carcinoma cell lines that secrete proteinase inhibitors *in vitro*. *Virchows Arch. B. Cell Pathol*., **57**, 157.

KARASHIMA, S., KATAOKA, H., ITOH, H., MARUYAMA, R. & KOONO, M. (1990). Prognostic significance of alpha-1-antitrypsin in early stage of colorectal carcinomas. *Int. J. Cancer*, **45**, 244.

KITTAS, C., ARONI, K., MATANI, A. & PAPADIMITRIOU, C.S. (1982*a*). Immunocytochemical demonstration of a1-antitrypsin and a1-antichymotrypsin in human gastrointestinal tract. *Hepatogastroenterology*, **29**, 275.

KITTAS, C., ARONI, K., KOTSIS, L. & PAPADIMITRIOU, C.S. (1982*b*). Distribution of lysozyme, a1-antichymotrypsin and a1-antitrypsin in adenocarcinomas of the stomach and large intestine. An immunohistochemical study. *Virchows Arch. A*., **398**, 139.

KOJ, A., CHUDZIK, J., POJDAK, W. & DUBLIN, A. (1972). The occurrence of common inhibitors of trypsin and of leukocyte neutral proteinase in human serum. *Biochim. Biophys. Acta*, **268**, 199.

KRUGLIANK, L., MEYER, P.R. & TAYLOR, C.R. (1986). The distribution of lysozyme, alpha-1-antitrypsin and alpha-1-antichymotrypsin in normal hematopoietic cells and in myeloid leukemias: an imunoperoxidase study on cytocentrifuge preparations, smears, and paraffin sections. *Am. J. Hematol*., **21**, 99.

LAURELL, C.B. & JEPPSSON, J.O. (1975). Protease inhibitor in plasma. II. alpha1-antitrypsin. In *The Plasma Proteins, Structure, Function, and Genetic Control*, Vol. I, Putnam, F.W. (ed.) p. 232. Academic Press: New York.

MIGNATTI, P., ROBBINS, E. & RIFKIN, D.B. (1986). Tumor invasion through the human amniotic membrane: requirement for a proteinase cascade. *Cell*, **47**, 487.

MCKEEHAN, W.L., SAKAGAMI, Y., HOSHI, H. & MCKEEHAN, K.A. (1986). Two apparent human endothelial cell growth factors from human hepatoma cells are tumor associated proteinase inhibitors. *J. Biol. Chem*., **261**, 5378.

MOUNTAIN, C.F. (1986). A new international staging system for lung cancer. *Chest*, **89**, 225.

PERLMUTTER, D.H., DANIELS, J.D., AUERBACH, H.S., SCHRYVER-KECSKEMETI, K.D., WINTER, H.S. & ALPERS, D.H. (1989). The alpha1-antitrypsin gene is expressed in a human intestinal epithelial cell line. *J. Biol. Chem*., **264**, 9485.

RAY, M.B. & DESMET, V.J. (1978). Immunohistochemical demonstration of alpha-1-antitrypsin in the islet cells of human pancreas. *Cell Tiss. Res*., **187**, 69.

REINTOFT, I. & HAGERSTRAND, I. (1979). Demonstration of alpha1-antitrypsin in hepatomas. *Arch. Pathol. Lab. Med*., **103**, 495.

REDELMAN, D. & HUDIG, D. (1980). The mechanism of cell-mediated cytotoxicity. I. Killing by murine cytotoxic T lymphocytes requires cell surface thiols and activated proteases. *J. Immunol*., **124**, 870.

RIMON, A., SHAMASH, Y. & SHAPIRO, B. (1966). The plasmin inhibitor of human plasma. IV. Its action on plasmin, trypsin, chymotrypsin and thrombin. *J. Biol. Chem*., **241**, 5102.

SAWAYA, R., ZUCCZRELLO, M. & HIGHSMITH, R. (1987). Alpha-1-antitrypsin in human brain tumors. *J. Neurosurg*., **67**, 258.

SOINI, Y. & MIETTINEN, M. (1989). Alpha-1-antitrypsin and lysozyme. Their limited significance in fibrohistiocytic tumors. *Am. J. Clin. Pathol*., **91**, 515.

TAHARA, E., ITO, H., TANIYAMA, K., YOKOZAKI, H. & HATA, J. (1984). Alpha1-antitrypsin, alpha1-antichymotrypsin, and alpha2-macroglobulin in human gastric carcinomas: a retrospective immunohistochemical study. *Hum. Pathol*., **15**, 957.

TRYGGVASON, K., HOYHTYA, M. & SALO, T. (1987). Proteolytic degradation of extracellular matrix in tumor invasion. *Biochim. Biophys. Acta*, **907**, 191.

TUTTLE, W.C. & JONES, R.K. (1975). Fluorescent antibody study of alpha-1-antitrypsin in adult human lung. *Am. J. Clin. Pathol*., **64**, 477.

WITTEKIND, CH., WACHNER, R., HENKE, W. & VON KLEIST, S. (1986). Localization of CEA, HCG, lysozome, alpha-1-antitrypsin, and alpha-1-antichymotrypsin in gastric cancer and prognosis. *Virchows Arch. A*., **409**, 715.

ZUCKER, S. (1988). A critical appraisal of the role of proteolytic enzymes in cancer invasion: emphasis on tumor surface proteinases. *Cancer Invest*., **6**, 219.